# PReS-FINAL-1014: Role of MHC class I overexpression on muscle biopsy of patients with juvenile dermatomyositis

**DOI:** 10.1186/1546-0096-11-S2-P11

**Published:** 2013-12-05

**Authors:** J Sanchez-Manubens, E Iglesias, C Jou, MI Gonzalez, R Bou, V Torrente-Segarra, S Ricart, J Calzada-Hernandez, J Anton

**Affiliations:** 1 Unit of Pediatric Rheumatology, Department of Pediatrics, Barcelona, Spain; 2 Department of Pathology, Hospital Saint Joan de Déu, Esplugues de Llobregat, Barcelona, Spain

## Introduction

Juvenile Dermatomyositis (JDM) is the most common idiopathic inflammatory myopathy in childhood, a systemic vasculopathy that usually affects skin and skeletal muscle but also can affect gastrointestinal tract and other organs. Diagnosis is based on Bohan and Peter's criteria and the goals of treatment include control of skin and muscle symptoms and prevention of disease complications. Stepwise aggressive treatment decreases JDM activity and improves long-term outcome.

Muscle involvement in JDM can be assessed by electromyography (EMG), magnetic resonance imaging (MRI) and/or muscle biopsy.

Muscle biopsy assesses the presence of lymphocytic inflammatory infiltrate, perifascicular atrophy, muscle fibers necrosis and it allows studying the overexpression of major histocompatibility complex (MHC) class I in the sarcolemma and sarcoplasm of the muscle cell. In healthy muscles, there is no MHC class I expression but in inflammatory myopathies there is a distinctive and generalized overexpression, not limited to the affected areas, it appears before the inflammatory infiltrate occurs and it is not modified by immunosuppressive treatment.

## Objectives

To assess MHC I overexpression on muscle biopsy of our patients with JDM.

## Methods

Descriptive retrospective review of our JDM patients with muscle biopsy at diagnosis between January 2000 and December 2011. In all cases, muscle biopsy techniques were performed with hematoxylin-eosin, trichrome, oxidative enzymes (NADH, SDH, COX and COX-SDH), neonatal myosin and MHC class I.

## Results

12 patients were included, 8 of them were girls (66%). All children had cutaneous and muscular clinical features at diagnosis. All MRI were pathological before treatment. EMG was performed in 9 patients, being altered in 8 of them (88%). Childhood Myositis Assessment Scale (CMAS) was done in 6 children with a median score of 30.5/52 (range 12-50/52). We found pathological muscle biopsy in 8 children (66%). Mild endomisial inflammation was found in 5 (41%) patients and moderate inflammation in 4 (33%). No vasculitis was found, but 7 (58%) patients had necrotic muscle fibers in biopsy. Immunohistochemical staining for MHC class I was positive in all cases, even in those children with normal muscle biopsy.

## Conclusion

MHC class I overexpression is present in muscle biopsy of all our patients with suspected JDM, even those with negative EMG or normal CMAS score. Although it is an invasive technique, muscle biopsy including MHC class I staining should be performed in all patients with suspected JDM in order to achieve more accurate diagnostic and establish an early and aggressive treatment.

## Disclosure of interest

None declared.

